# Supplementary material for: BTK, NUTM2A, and PRPF19 Are Novel KMT2A Partner Genes in Childhood Acute Leukemia
Source: Biomedicines. 2021 Jul 30;9(8):924. doi: 10.3390/biomedicines9080924 (PMC8391293; doi:10.3390/biomedicines9080924)
Supplement: Supplementary file 1 [file biomedicines-09-00924-s001.zip › biomedicines-1299339-supplementary.pdf]

biomedicines

Supplementary materials

BTK, NUTM2A, and *PRPF19* are Novel *KMT2A* Partner Genes in Childhood Acute Leukemia

Elena Zerkalenkova [1,*], Svetlana Lebedeva [1], Aleksandra Borkovskaia [1], Olga Soldatkina [1], Olga Plekhanova [2], Grigory Tsaur [2], Michael Maschan [1], Aleksey Maschan [1], Galina Novichkova [1] and Yulia Olshanskaya [1]

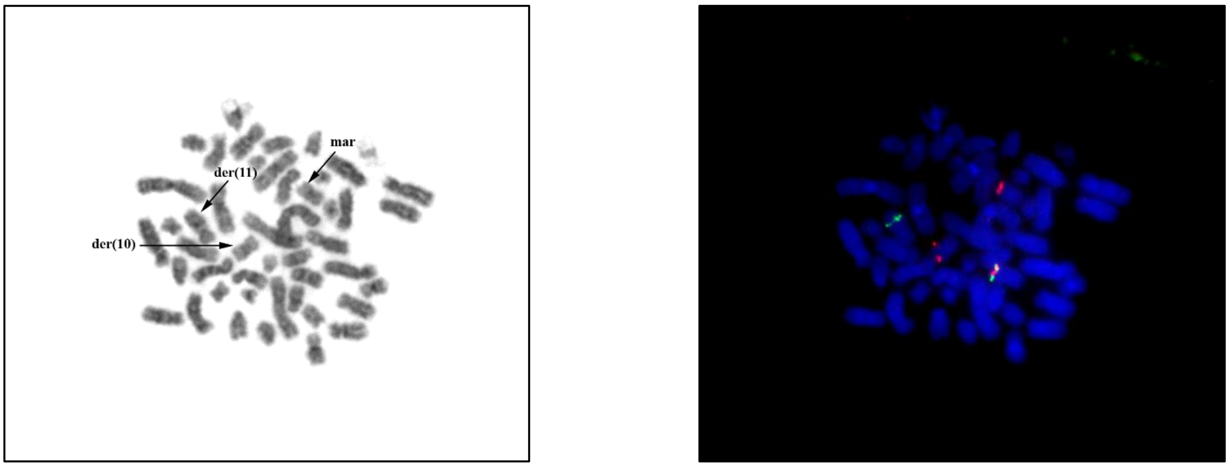

(**a**) (**b**)

Figure S1. *KMT2A-NUTM2A* fusion gene in T-ALL with t(10;11)(q22;q23.3): (**a**) 47,XX,t(10;11)(q22;q23),+mar karyotype by G-banding, rearranged chromosomes 10 and 11 and a marker chromosome are marked with arrows; (**b**) *KMT2A* gene rearrangement by FISH with the Kreatech ON KMT2A break-apart probe (Leica) on the same metaphase plate.

Table S1. Validation primers for novel *KMT2A* fusion genes and transcripts.

Rearrangement	Fusion Gene	Fusion Transcript
KMT2A-BTK	MLL-F1<br>CCCAAGTATCCCTGTAAAACAAAAA<br>BTK-R<br>TCTACCAGAATTGCCATGATTTGG	MLL-F8<br>CGCCTCAGCCACCTACTACAG<br>BTK-R<br>CACGGTCAAGAGAAACAGGCG
KMT2A-NUTM2A	MLL-F2<br>ATTACCAAATCAGGAAATTAACACACTGG<br>NUTM2A-R<br>TTTCTGAGGACTTGATCTTTTACCATGGGG<br>NUTM2A-F<br>AAATCCTGTATCAAAGCCACGTGTGAG<br>MLL-R<br>CCAAGTCTGTTGTGAGCCCTTCCA	MLL-F11<br>CAGAAAATGTGTGGGAGATGGG<br>NUTM2A-R<br>CACTGACCACCGCTGTCTGG
KMT2A-PRPF19	MLL-F8<br>CGCCTCAGCCACCTACTACAG<br>PRPF19-R<br>GAAGCGCGTTGTAAACCAATAAG	MLL-F8<br>CGCCTCAGCCACCTACTACAG<br>PRPF19-R1<br>GAGGCTTGGGCCGGATTGGG
